# Genome resequencing and bioinformatic analysis of SNP containing candidate genes in the autoimmune vitiligo Smyth line chicken model

**DOI:** 10.1186/1471-2164-15-707

**Published:** 2014-08-23

**Authors:** Hyeon-Min Jang, Gisela F Erf, Kaylee C Rowland, Byung-Whi Kong

**Affiliations:** Department of Poultry Science, Center of Excellence for Poultry Science, University of Arkansas, POSC O-404, 1260 West Maple, Fayetteville, AR 72701 USA

## Abstract

**Background:**

The Smyth line (SL) chicken is the only animal model for autoimmune vitiligo that spontaneously displays all clinical and biological manifestations of the human disorder. To understand the genetic components underlying the susceptibility to develop SL vitiligo (SLV), whole genome resequencing analysis was performed in SLV chickens compared with non-vitiliginous parental Brown line (BL) chickens, which maintain a very low incidence rate of vitiligo.

**Results:**

Illumina sequencing technology and reference based assembly on Red Jungle Fowl genome sequences were used. Results of genome resequencing of pooled DNA of each 10 BL and SL chickens reached 5.1x and 7.0x coverage, respectively. The total number of SNPs was 4.8 and 5.5 million in BL and SL genome, respectively. Through a series of filtering processes, a total of ~1 million unique SNPs were found in the SL alone. Eventually of the 156 reliable marker SNPs, which can induce non-synonymous-, frameshift-, nonsense-, and no-start mutations in amino acid sequences in proteins, 139 genes were chosen for further analysis. Of these, 14 randomly chosen SNPs were examined for SNP verification by PCR and Sanger sequencing to detect SNP positions in 20 BL and 70 SL chickens. The results of the analysis of the 14 SNPs clearly showed differential frequencies of nucleotide bases in the SNP positions between BL and SL chickens. Bioinformatic analysis showed that the 156 most reliable marker SNPs included genes involved in dermatological diseases/conditions such as ADAMTS13, ASPM, ATP6V0A2, BRCA2, COL12A1, GRM5, LRP2, OBSCN, PLAU, RNF168, STAB2, and XIRP1. Intermolecular gene network analysis revealed that candidate genes identified in SLV play a role in networks centered on protein kinases (MAPK, ERK1/2, PKC, PRKDC), phosphatase (PPP1CA), ubiquitinylation (UBC) and amyloid production (APP).

**Conclusions:**

Various potential genetic markers showing amino acid changes and potential roles in vitiligo development were identified in the SLV chicken through genome resequencing. The genetic markers and bioinformatic interpretations of amino acid mutations found in SLV chickens may provide insight into the genetic component responsible for the onset and the progression of autoimmune vitiligo and serve as valuable markers to develop diagnostic tools to detect vitiligo susceptibility.

**Electronic supplementary material:**

The online version of this article (doi:10.1186/1471-2164-15-707) contains supplementary material, which is available to authorized users.

## Background

Vitiligo is an acquired hypomelanotic disorder characterized by circumscribed depigmented macules in the skin resulting from the loss of melanocytes. Autoimmunity has been identified as the major etiological factor in vitiligo, although many other factors including infections, stress, neural abnormalities, aberrant melanocyte function, and genetic susceptibility have been implicated [1]. The Smyth line (SL) chicken is the only animal model for autoimmune vitiligo that spontaneously displays all clinical and biological manifestations of the human disorder [2, 3]. Like other autoimmune diseases, SL vitiligo (SLV) is multi-factorial in nature and involves the interplay of genetic, immune system, and environmental-factors. SLV susceptibility is manifested in part in an inherent melanocyte defect and loss of melanocytes is due to melanocyte-specific cell-mediated and humoral immune activities [2, 3].

Recent genome wide association studies (GWAS) in humans to understand the role of genetic components in a variety of autoimmune diseases including vitiligo have identified hundreds of loci harboring risk alleles [4]. Several GWAS results identified vitiligo susceptible loci in human populations [5–10]. However, most susceptible loci identified by GWAS results were found in regulatory regions of gene expression, therefore the identified associations were not sufficient to identify the causal gene or deduce alterations caused by risk variants, which generally do not induce profound changes to genes (e.g. coding sequence changes, deletions, or duplications). Recently, the encyclopedia of DNA elements (ENCODE) of mammalian species suggested that ~90% of disease associated genetic variations in human lie in noncoding regions, while only ~10% of variations in coding regions were causative mutations associated with human disease [11, 12]. Nevertheless, the identification of potential coding mutations that alter protein functionalities is a prerequisite process to understand disease etiology. Moreover, the functional study of candidate genetic risk factors is almost impossible without appropriate model systems.

The SL chicken is an excellent model to conduct a functional verification study of candidate genes that underlie genetic susceptibility for vitiligo due to the tractable, definite phenotype, the high vitiligo incidence in the population (80-90%), the feasibility of in vivo characterization and the relatively short generation time. Recently, microarray analysis examined global gene expression during SLV development and provided comprehensive information at the transcriptome level that supported the multifactorial etiology of vitiligo [13]. In this study, whole genome resequencing analysis using an Illumina platform was performed to more deeply investigate the genetic aspects of SLV expression in comparison with the parental Brown line (BL) of chickens from which the SL was originally derived. BL chickens retain vitiligo susceptibility but with a very low (0 – 2%) incidence rate of vitiligo development [2, 3], although none of the BL chickens used in this study had vitiligo. Millions of single nucleotide polymorphisms (SNP) were identified by genome resequencing and only potentially causal genes containing non-synonymous mutations that can induce amino acid changes in proteins were focused on in this study.

## Results and discussion

### Genome resequencing for BL and SL chickens

**Figure 1:**
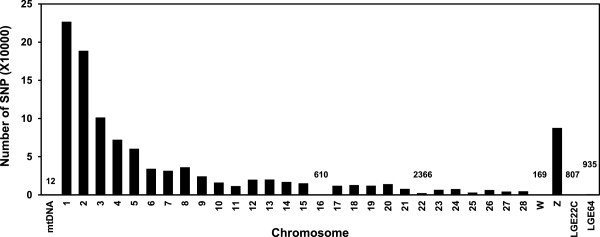
**Number of unique SNPs per chromosome found in vitiliginous SL chickens compared to non-vitiliginous BL chickens.** Numbers are indicated for bars not clearly visible.

**Figure 2:**
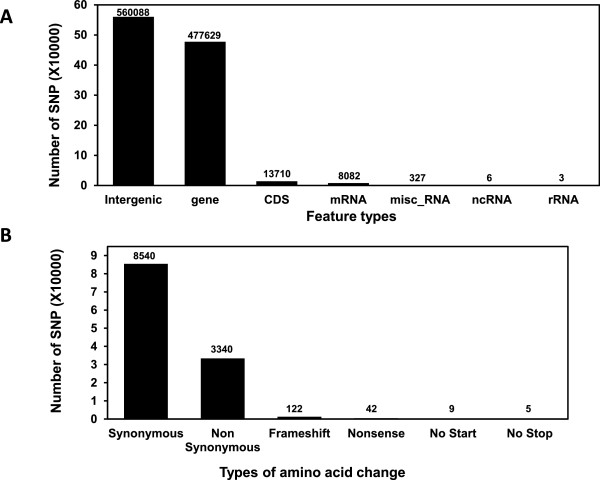
**Summary of SNPs in SLV. A)** Number of SNPs categorized by chromosomal region in SL chickens. **B)** Number of SNPs categorized by type of amino acid sequence changes.

Genome sequencing of pooled DNA from 10 non-vitiliginous BL and SL chickens each with confirmed SLV produced ~63 and 89 million sequence reads of 200 bp, respectively (Table 1). Of those, ~80% of the reads were used for sequence alignment, while 20% of sequence reads were not aligned. Therefore, genome coverage for BL and SL reached 5.1x and 7.0x, respectively, of the Red Jungle Fowl chicken genome. The total number of SNPs was 4.8 and 5.5 million (~0.5% of template genome) for the BL and SL genome, respectively. The large number of SNPs per examined chicken line was based on data of at least 2 read coverage depths (number of read counts per nucleotide location). Most SNPs were found on the larger chromosomes (Chr), including Chr 1 through 5 and Z (sex chromosome) (data not shown). To identify genetic biomarkers that are responsible for the incidence of SLV, unique SNPs that are found in SL only were selected by removing SNPs that overlapped with those found in the parental BL. Then, mutations with ≥75% SNP rates were chosen as reliable marker SNPs. Since the objective of this study was to identify mutational SNPs uniquely found in SL compared to the parental BL, the filtering process used did not involve a typical SNP calling and filtering method based on quality score (Q call column in Additional file 1: Table S1) [14]. Instead, SNP filtering was conducted by removing overlapping SNPs found in both BL and SL, and applying fixed %SNP rates (≥75%) as described in Methods. As a result, a total of ~1 million unique SNPs were identified throughout the SL chicken genome (Figure 1). Over 100,000 SNPs were found in Chr 1, 2, 3, and Z while Chr 32 did not contain any unique SNPs for SL. When SNPs were grouped by the feature types of chromosome regions, ~50% of SNPs were in the intergenic (heterochromatic) regions and 13,710 SNPs were found in CDS sequences (protein coding regions) (Figure 2). Most genes containing SNPs in regulatory regions, not in CDS regions, identified by human GWAS studies were also observed to contain unique SNPs in the current SL study (data not shown). Around 60% of SL SNPs in protein coding CDS regions were synonymous mutations that did not induce amino acid changes. To identify potentially causal mutations that induce protein coding alterations, SNP analysis focused on SNPs leading to changes in amino acid sequences. Using this approach, a total of 3518 SNPs were identified that could induce non-synonymous-, frameshift-, nonsense-, and no-start-changes in the CDS region (Figure 2 and Additional file 1: Table S1), suggesting that the 3518 SNPs are part of the genetic components in functional protein coding regions that may drive the high incidence rate of SLV. Of the 3518 candidate SNPs that are associated with amino acid changes, SNPs showing ≥10 read depths (considered to be more reliable candidate genetic markers) were chosen for the further analysis. Using this approach, 195 SNPs remained (data not shown). To reduce false positives due to possible errors in the assembly process, re-scanning of each SNP position for the 195 potentially more reliable protein coding SNPs was conducted using the Seqman-Pro viewer program. This process yielded 156 more reliable SNPs that were chosen as candidate marker SNPs for further analysis (Table 2).

**Table 1:** **Results of Illumina sequencing and assembly**

Line	Reads numbers	Total # of reads used for alignment	Total # of reads not aligned	Coverage	Total # of SNP
BL (P) ^1^	62,764,368	50,536,831	9,480,794	5.1x	4,846,132
SL^2^	88,525,266	69,886,110	14,225,577	7.0x	5,465,994

^1^(P) means the parental line, no vitiligo.

^2^ SL, with vitiligo.

**Table 2:** **The 156 reliable marker SNPs that induced amino acid changes showing** **≥10 read depths**

Contig ID	c	Ref Pos	Ref base	Called base	Impact	SNP%	Gene symbol	DNA change	AA Change	Depth	A Cnt	C Cnt	G Cnt	T Cnt	Del
NC_006088	1	12587537	C	T	N-Syn	0.80	FGL2	c.368C > T	T123I	10	0	-	0	8	0
NC_006088	1	24296415	G	C	N-Syn	1.00	CTTNBP2	c.2600G > C	R867P	12	0	12	-	0	0
NC_006088	1	33755434	G	A	N-Syn	0.91	TBC1D30	c.304G > A	V102I	11	10	0	-	0	0
NC_006088	1	34386453	G	A	N-Syn	1.00	HELB	c.2342G > A	G781E	12	12	0	-	0	0
NC_006088	1	34386455	C	T	N-Syn	1.00	HELB	c.2344C > T	R782W	12	0	-	0	12	0
NC_006088	1	45803846	A	G	N-Syn	1.00	LOC417921	c.1457A > G	Q486R	11	-	0	11	0	0
NC_006088	1	45804772	T	A	N-Syn	0.92	LOC417921	c.1700 T > A	I567K	13	12	1	0	-	0
NC_006088	1	54936701	G	T	N-Syn	0.80	STAB2	c.6155G > T	A2052D	10	0	0	-	8	0
NC_006088	1	67320079	C	C|A	N-Syn	0.77	CASC1	c.[949C > C] + [949C > A]	V317V, V317F	13	10	-	0	0	0
NC_006088	1	82856208	G	T	N-Syn	1.00	CD200R1	c.197G > T	R66L	11	0	0	-	11	0
NC_006088	1	88049869	C	T	N-Syn	0.90	LOC418423	c.229C > T	V77I	10	0	-	0	9	0
NC_006088	1	88157238	C	T	N-Syn	1.00	CGGBP1	c.428C > T	R143Q	10	0	-	0	10	0
NC_006088	1	105941417	T	C	N-Syn	0.90	SETD4	c.1186 T > C	I396V	10	0	9	0	-	0
NC_006088	1	107377588	C	G	N-Syn	0.92	LCA5L	c.1331C > G	G444A	13	0	-	12	0	0
NC_006088	1	108406755	T	T|C	N-Syn	0.77	LOC770616	c.[37 T > T] + [37 T > C]	M13M, M13V	13	0	10	0	-	0
NC_006088	1	108764081	C	T	N-Syn	0.91	UBASH3A	c.1144C > T	L382F	11	0	-	0	10	0
NC_006088	1	109036880	G	T	N-Syn	1.00	CBS	c.1492G > T	Q498K	10	0	0	-	10	0
NC_006088	1	112264749	A	G	N-Syn	1.00	OTC	c.5A > G	L2P	12	-	0	12	0	0
NC_006088	1	112303781	A	G	N-Syn	1.00	RPGR	c.2359A > G	I787V	13	-	0	13	0	0
NC_006088	1	112303950	G	A	N-Syn	1.00	RPGR	c.2528G > A	G843E	10	10	0	-	0	0
NC_006088	1	121737171	G	A	N-Syn	1.00	FANCB	c.1988G > A	R663K	12	12	0	-	0	0
NC_006088	1	121737188	C	A	N-Syn	1.00	FANCB	c.2005C > A	L669M	12	12	-	0	0	0
NC_006088	1	133392696	A	G	N-Syn	1.00	MFSD9	c.790A > G	F264L	11	-	0	11	0	0
NC_006088	1	137555484	C	T|C	N-Syn	0.79	MYO16	c.[1232C > T] + [1232C > C]	T411T, T411I	14	0	-	0	11	0
NC_006088	1	138584430	C	T	N-Syn	0.90	ANKRD10	c.551C > T	R184H	10	0	-	0	9	0
NC_006088	1	152732014	A	G	N-Syn	1.00	RNF219	c.794A > G	Q265R	10	-	0	10	0	0
NC_006088	1	153202063	C	T	N-Syn	1.00	SLAIN1	c.1486C > T	G496S	11	0	-	0	11	0
NC_006088	1	166907615	G	C	N-Syn	0.90	NUFIP1	c.925G > C	Q309E	10	0	9	-	0	0
NC_006088	1	169375045	A	G	N-Syn	0.91	SERPINE3	c.254A > G	H85R	11	-	0	10	0	0
NC_006088	1	173852809	T	C	N-Syn	1.00	BRCA2	c.355 T > C	T119A	10	0	10	0	-	0
NC_006088	1	176891377	A	G	N-Syn	1.00	SACS	c.6235A > G	I2079V	11	-	0	11	0	0
NC_006088	1	177772554	C	A	N-Syn	0.86	EFHA1	c.659C > A	A220D	14	12	-	0	0	0
NC_006088	1	177772568	G	A	N-Syn	0.86	EFHA1	c.673G > A	V225I	14	12	0	-	0	0
NC_006088	1	178541726	G	T	N-Syn	0.92	CENPJ	c.2041G > T	V681L	12	0	0	-	11	0
NC_006088	1	187399843	G	A	N-Syn	0.90	GRM5	c.2842G > A	A948T	10	9	0	-	0	0
NC_006088	1	194954389	A	G	N-Syn	1.00	RNF169	c.992A > G	V331A	10	-	0	10	0	0
NC_006089	2	2046630	C	T	N-Syn	1.00	CCDC13	c.860C > T	S287N	12	0	-	0	12	0
NC_006089	2	2059597	T	C	N-Syn	1.00	CCDC13	c.209 T > C	D70G	11	0	11	0	-	0
NC_006089	2	2141930	A	G	N-Syn	0.90	OBSCN	c.17225A > G	I5742T	10	-	0	9	0	0
NC_006089	2	2155796	T	C	N-Syn	1.00	OBSCN	c.14875 T > C	N4959D	11	0	11	0	-	0
NC_006089	2	4669968	C	T	N-Syn	0.90	DLEC1	c.1592C > T	A531V	10	0	-	0	9	0
NC_006089	2	4948035	C	T	N-Syn	1.00	XIRP1	c.4696C > T	A1566T	11	0	-	0	11	0
NC_006089	2	7746258	C	-	Frameshift	1.00	LOC100859401	c.388C > -	Y129fs	10	0	-	0	0	10
NC_006089	2	20725367	C	A	N-Syn	1.00	DBF4	c.1114C > A	P372T	11	11	-	0	0	0
NC_006089	2	21576571	C	T	N-Syn	0.90	C2H7orf63	c.962C > T	T321M	10	0	-	0	9	0
NC_006089	2	23984791	G	A	N-Syn	0.90	PON2	c.521G > A	P174L	10	9	0	-	0	0
NC_006089	2	26789159	T	C	N-Syn	1.00	VWDE	c.2678 T > C	N893S	11	0	11	0	-	0
NC_006089	2	42356129	C	T	N-Syn	1.00	LOC100857506	c.757C > T	P253S	12	0	-	0	12	0
NC_006089	2	43749632	C	T	N-Syn	1.00	TGM4	c.160C > T	A54T	10	0	-	0	10	0
NC_006089	2	47501151	C	A	N-Syn	1.00	SEPT7	c.764C > A	G255V	10	10	-	0	0	0
NC_006089	2	48103938	C	T	N-Syn	1.00	BMPER	c.1957C > T	V653I	11	0	-	0	11	0
NC_006089	2	67476715	T	A	N-Syn	1.00	MYLK4	c.2071 T > A	I691L	12	12	0	0	-	0
NC_006089	2	79168177	C	T	N-Syn	1.00	MTRR	c.2027C > T	R676K	11	0	-	0	11	0
NC_006089	2	96844408	T	G	N-Syn	1.00	CEP192	c.3655 T > G	K1219Q	11	0	0	11	-	0
NC_006089	2	107668292	C	G	N-Syn	1.00	PRKDC	c.3938C > G	C1313S	10	0	-	10	0	0
NC_006089	2	107767247	A	G	N-Syn	0.91	LOC421108	c.77A > G	N26S	11	-	0	10	0	0
NC_006089	2	115050472	C	A	N-Syn	1.00	CSPP1	c.1303C > A	H435N	10	10	-	0	0	0
NC_006089	2	120054459	T	G	N-Syn	1.00	FAM164A	c.605 T > G	V202G	10	0	0	10	-	0
NC_006089	2	126210593	G	A	N-Syn	0.80	C2H8orf38	c.730G > A	V244I	10	8	0	-	0	0
NC_006089	2	148002272	C	T	N-Syn	1.00	TOP1MT	c.1149C > T	M383I	10	0	-	0	10	0
NC_006090	3	18177273	C	T	N-Syn	1.00	EPRS	c.778C > T	H260Y	10	0	-	0	10	0
NC_006090	3	18177438	T	C	N-Syn	1.00	EPRS	c.943 T > C	C315R	11	0	11	0	-	0
NC_006090	3	19964716	A	T	N-Syn	0.90	USH2A	c.9152A > T	E3051V	10	-	0	0	9	0
NC_006090	3	20371026	T	C	N-Syn	0.92	CENPF	c.2726 T > C	E909G	13	0	12	0	-	0
NC_006090	3	20377057	T	T|A	N-Syn	0.75	CENPF	c.[1411 T > T] + [1411 T > A]	N471N, N471Y	12	9	0	0	-	0
NC_006090	3	28850511	A	G	N-Syn	1.00	LOC100858112	c.586A > G	F196L	10	-	0	10	0	0
NC_006090	3	47388200	G	G|A	N-Syn	0.77	ZC3H12D	c.[998G > G] + [998G > A]	T333T, T333M	13	10	0	-	0	0
NC_006090	3	48575253	C	T	N-Syn	0.90	SYNE1	c.11674C > T	A3892T	10	0	-	0	9	0
NC_006090	3	48577869	C	T	N-Syn	0.93	SYNE1	c.11335C > T	D3779N	14	0	-	0	13	0
NC_006090	3	54551285	T	C	N-Syn	1.00	PDE7B	c.868 T > C	T290A	11	0	11	0	-	0
NC_006090	3	66462814	C	T	N-Syn	1.00	LOC421765	c.1076C > T	R359Q	12	0	-	0	12	0
NC_006090	3	66468226	G	T	N-Syn	1.00	LOC421765	c.334G > T	Q112K	10	0	0	-	10	0
NC_006090	3	75929093	G	A	N-Syn	0.93	ZNF292	c.7835G > A	T2612I	14	13	0	-	0	0
NC_006090	3	80234352	G	A	N-Syn	1.00	COL12A1	c.2659G > A	A887T	10	10	0	-	0	0
NC_006091	4	4124961	C	T	Nonsense	1.00	DDX26B	c.151C > T	Q51.	11	0	-	0	11	0
NC_006091	4	9883806	G	A	N-Syn	0.91	SLITRK4	c.1715G > A	R572K	11	10	0	-	0	0
NC_006091	4	49489771	G	T	N-Syn	0.91	RUFY3	c.1588G > T	A530S	11	0	0	-	10	0
NC_006092	5	3898544	C	T	N-Syn	0.80	KIF18A	c.1567C > T	D523N	10	0	-	0	8	0
NC_006092	5	8035275	A	G	N-Syn	1.00	LOC100859209	c.79A > G	C27R	11	-	0	11	0	0
NC_006092	5	20708767	A	G	N-Syn	1.00	API5	c.506A > G	K169R	10	-	0	10	0	0
NC_006092	5	21158570	-	A	Frameshift	0.90	LOC770458	c.105- > A	A35fs	10	9	0	0	0	1
NC_006092	5	22076516	T	C	N-Syn	1.00	C1QTNF4	c.230 T > C	I77T	11	0	11	0	-	0
NC_006092	5	38183079	C	T	N-Syn	0.83	LOC100859479	c.82C > T	P28S	12	0	-	0	10	0
NC_006092	5	44443744	G	G|C	N-Syn	0.75	KIAA1409	c.[2076G > G] + [2076G > C]	Q692H, Q692Q	12	0	9	-	0	0
NC_006092	5	44443754	G	A	N-Syn	0.80	KIAA1409	c.2086G > A	E696K	10	8	0	-	0	0
NC_006092	5	44443755	A	T	N-Syn	0.80	KIAA1409	c.2087A > T	E696V	10	-	0	0	8	0
NC_006092	5	45296293	G	G|A	N-Syn	0.75	C5H14orf49	c.[596G > G] + [596G > A]	A199A, A199V	12	9	0	-	0	0
NC_006092	5	50316949	A	G	N-Syn	1.00	APOPT1	c.440A > G	H147R	12	-	0	12	0	0
NC_006093	6	4563260	G	C	N-Syn	0.90	BMS1	c.1666G > C	L556V	10	0	9	-	0	0
NC_006093	6	15062678	A	G	N-Syn	0.80	PLAU	c.925A > G	W309R	10	-	0	8	0	0
NC_006093	6	25651167	G	A	N-Syn	1.00	RBM20	c.1687G > A	D563N	10	10	0	-	0	0
NC_006093	6	30617911	C	T	N-Syn	0.80	ATE1	c.518C > T	G173D	10	0	-	0	8	0
NC_006093	6	32898174	T	C	N-Syn	1.00	MKI67	c.2414 T > C	M805T	10	0	10	0	-	0
NC_006094	7	9605730	T	C	N-Syn	1.00	ANKRD44	c.584 T > C	K195R	15	0	15	0	-	0
NC_006094	7	18282262	G	G|A	N-Syn	0.75	LRP2	c.[3775G > G] + [3775G > A]	A1259T, A1259A	12	9	0	-	0	0
NC_006094	7	21828274	A	G|A	N-Syn	0.75	CCDC108	c.[2902A > G] + [2902A > A]	N968N, N968D	12	-	0	9	0	0
NC_006094	7	25803714	T	G	N-Syn	0.80	IQCB1	c.952 T > G	T318P	10	0	0	8	-	0
NC_006095	8	1373656	G	A	N-Syn	1.00	CAMSAP1L1	c.1955G > A	A652V	12	12	0	-	0	0
NC_006095	8	2290270	A	G	N-Syn	1.00	LOC100859900	c.65A > G	L22P	13	-	0	13	0	0
NC_006095	8	2506276	C	T	N-Syn	1.00	CRB1	c.1814C > T	S605N	12	0	-	0	12	0
NC_006095	8	2612732	T	C	N-Syn	1.00	ASPM	c.6907 T > C	C2303R	11	0	11	0	-	0
NC_006095	8	7596287	G	T	N-Syn	0.91	NCF2	c.1234G > T	P412T	11	0	0	-	10	0
NC_006095	8	8053190	G	C	N-Syn	0.81	LOC768407	c.875G > C	G292A	16	0	13	-	0	0
NC_006095	8	8188888	T	A	N-Syn	0.82	LOC768392	c.512 T > A	E171V	11	9	0	0	-	0
NC_006095	8	12666139	G	G|A	N-Syn	0.75	ABCA4	c.[4678G > G] + [4678G > A]	V1560I, V1560V	12	9	0	-	0	0
NC_006095	8	23585119	C	G	N-Syn	0.93	LRP8	c.583C > G	G195R	14	0	-	13	0	0
NC_006096	9	800651	G	G|A	N-Syn	0.75	LOC424748	c.[5G > G] + [5G > A]	R2K, R2R	12	9	0	-	0	0
NC_006096	9	2123518	A	G	N-Syn	1.00	YEATS2	c.602A > G	N201S	11	-	0	11	0	0
NC_006096	9	4127341	G	A	N-Syn	1.00	GAL3ST4	c.505G > A	A169T	10	10	0	-	0	0
NC_006096	9	4217231	A	G	N-Syn	0.92	RNF168	c.806A > G	D269G	13	-	0	12	0	0
NC_006096	9	15175477	T	G	N-Syn	1.00	CAPN10	c.1660 T > G	T554P	11	0	0	11	-	0
NC_006096	9	21419266	C	A	N-Syn	0.82	SPTSSB	c.44C > A	P15Q	11	9	-	0	0	0
NC_006097	10	3986458	T	C	N-Syn	0.82	VPS13C	c.3727 T > C	Y1243H	11	0	9	0	-	0
NC_006099	12	9103119	G	A	N-Syn	1.00	COPG	c.1771G > A	A591T	12	12	0	-	0	0
NC_006099	12	15133837	T	C	N-Syn	1.00	LOC100858715	c.89 T > C	V30A	11	0	11	0	-	0
NC_006099	12	19738746	C	T	N-Syn	1.00	NR2C2	c.1061C > T	S354N	10	0	-	0	10	0
NC_006100	13	2703509	A	G|A	N-Syn	0.77	LOC769940	c.[2875A > G] + [2875A > A]	S959P, S959S	13	-	0	10	0	0
NC_006100	13	2703511	A	G|A	N-Syn	0.77	LOC769940	c.[2873A > G] + [2873A > A]	F958S, F958F	13	-	0	10	0	0
NC_006100	13	11337736	C	G	N-Syn	0.90	GEMIN5	c.849C > G	H283Q	10	0	-	9	0	0
NC_006100	13	11349274	A	G	N-Syn	0.83	GEMIN5	c.3739A > G	S1247G	12	-	0	10	0	0
NC_006101	14	5552664	G	A	N-Syn	0.82	TEKT4	c.1192G > A	V398I	11	9	0	-	0	0
NC_006101	14	5975913	T	C	N-Syn	0.93	CHTF18	c.1934 T > C	E645G	14	0	13	0	-	0
NC_006101	14	6002290	G	G|C	N-Syn	0.75	MSLN	c.[774G > G] + [774G > C]	S258S, S258R	12	0	9	-	0	0
NC_006101	14	7697077	C	T	N-Syn	0.80	ABCC6	c.1213C > T	V405M	10	0	-	0	8	0
NC_006101	14	13378210	T	C	N-Syn	0.93	C14H16orf89	c.68 T > C	D23G	14	1	13	0	-	0
NC_006102	15	3706306	T	C	N-Syn	1.00	GLT1D1	c.968 T > C	Q323R	10	0	10	0	-	0
NC_006102	15	4915706	T	C	N-Syn	0.90	ATP6V0A2	c.1246 T > C	I416V	10	0	9	0	-	0
NC_006102	15	4926428	G	A	N-Syn	1.00	LOC100857705	c.1552G > A	R518C	10	10	0	-	0	0
NC_006102	15	5060494	G	A	N-Syn	1.00	MPHOSPH9	c.755G > A	R252K	14	14	0	-	0	0
NC_006102	15	5695994	G	A	N-Syn	0.90	WDR66	c.1690G > A	D564N	10	9	0	-	0	0
NC_006102	15	6317399	C	T	N-Syn	1.00	C15H12orf51	c.7777C > T	D2593N	10	0	-	0	10	0
NC_006102	15	6633596	T	C	N-Syn	1.00	USP30	c.488 T > C	I163T	13	0	13	0	-	0
NC_006102	15	8096384	T	C	N-Syn	1.00	DDX51	c.1493 T > C	M498T	11	0	11	0	-	0
NC_006102	15	8693805	G	C	N-Syn	1.00	RTDR1	c.118G > C	V40L	10	0	10	-	0	0
NC_006104	17	6506143	T	C	N-Syn	0.87	SETX	c.4564 T > C	I1522V	15	0	13	0	-	0
NC_006104	17	6581087	G	A	N-Syn	1.00	C17H9orf171	c.682G > A	D228N	12	12	0	-	0	0
NC_006104	17	6922987	A	G	N-Syn	1.00	ADAMTS13	c.3103A > G	I1035V	12	-	0	12	0	0
NC_006104	17	7858127	A	T	N-Syn	1.00	SEC16A	c.4079A > T	H1360L	15	-	0	0	15	0
NC_006104	17	7905701	G	C	N-Syn	1.00	SNAPC4	c.2609G > C	G870A	11	0	11	-	0	0
NC_006105	18	1082307	C	T	N-Syn	0.90	DNAH9	c.6700C > T	P2234S	10	0	-	0	9	0
NC_006105	18	1111244	G	A	N-Syn	0.80	DNAH9	c.8491G > A	V2831M	10	8	0	-	0	0
NC_006105	18	6605385	C	G	N-Syn	1.00	ATAD5	c.3652C > G	P1218A	11	0	-	11	0	0
NC_006105	18	6605422	G	A	N-Syn	1.00	ATAD5	c.3689G > A	R1230K	10	10	0	-	0	0
NC_006105	18	6967029	C	T	N-Syn	1.00	C18H17orf58	c.1018C > T	L340F	12	0	-	0	12	0
NC_006106	19	5858876	T	C	N-Syn	1.00	ERAL1	c.1186 T > C	M396V	14	0	14	0	-	0
NC_006106	19	6213750	G	C	N-Syn	1.00	SLC6A4	c.242G > C	A81G	10	0	10	-	0	0
NC_006106	19	7495965	C	G	N-Syn	0.82	BRIP1	c.1718C > G	P573R	11	0	-	9	0	0
NC_006107	20	12338968	T	G	N-Syn	0.83	CASS4	c.1169 T > G	N390T	12	0	0	10	-	0
NC_006107	20	12696859	T	A	N-Syn	1.00	CYP24A1	c.878 T > A	L293Q	12	12	0	0	-	0
NC_006108	21	2336588	G	A	Nonsense	0.80	TAS1R3	c.1108G > A	R370.	10	8	0	-	0	0
NC_006108	21	3215527	C	T	N-Syn	0.92	ENO1	c.1265C > T	R422H	12	0	-	0	11	0
NC_006108	21	4657639	G	A	N-Syn	1.00	KIAA0090	c.2716G > A	R906C	11	11	0	-	0	0
NC_006110	23	5163924	T	C	N-Syn	1.00	ZBTB8A	c.232 T > C	F78L	10	0	10	0	-	0
NC_006112	25	1498400	G	A	N-Syn	1.00	HORMAD1	c.844G > A	A282T	11	11	0	-	0	0
NC_006113	26	4679877	G	A	N-Syn	1.00	LOC768535	c.1097G > A	P366L	10	10	0	-	0	0
NC_006127	Z	71529316	G	A	N-Syn	1.00	DMXL1	c.4394G > A	A1465V	11	11	0	-	0	0

Contig ID, chromosome (Chr) numbers, reference position (Ref Pos), reference base (Ref Base), called (SNP) base, impact (kinds of protein mutation), SNP%, feature name (gene name), DNA change, amino acid (AA) change, Depths, and five columns for SNP counts (cnts) are indicated.

### SNP validation using PCR and Sanger sequencing

Since pooled DNA samples of 10 chickens for each line were used for genome sequencing, individual SNPs were subjected to the verification process with larger bird populations. For this, 14 SNPs were randomly chosen from the 156 candidate marker SNPs showing ≥10 read depths and were subjected to SNP verification analysis using PCR and Sanger sequencing to detect SNP positions with larger numbers of birds; specifically 20 non-vitiliginous BL chickens and 70 SL chickens exhibiting vitiligo. The results clearly showed differential frequencies of nucleotide bases in the 14 SNP positions between BL and SL chickens (Table 3). Thus, the 156 SNPs known to induce amino acid changes can become potential genetic biomarkers for vitiligo in SL chickens.

**Table 3:** **Verification of 14 SNPs using PCR and Sanger sequencing in larger numbers of non-vitiliginous parental BL (20) vs. vitiliginous SL (70) chickens**

Ch	Ref Pos	Ref base	Called base	Impact	SNP%	Feature name	Protein change	Results of larger population	
								BL (20 birds)	SL (70 birds)
2	7746258	CT	-	FS	1	LOC100859401	Y129fs	**All_CT**	**All_Del**
5	21158570	-	A	FS	0.9	LOC770458	A35fs	**Ins_A_30%**	**Ins_A_100%**
9	21419266	C	A	N-Syn	0.82	SPTSSB [1]	P15Q	**C:A = 3:1**	**C:A = 1:4**
1	54936701	G	T	N-Syn	0.8	STAB2	A2052D	**All_G**	**G:T = 1:1**
3	48575253	C	T	N-Syn	0.9	SYNE1	A3892T	**C:T = 10:1**	**C:T = 1:10**
3	18177438	T	C	N-Syn	1	EPRS	C315R	**T:C = 4:1**	**T:C = 1:7**
1	105941417	T	C	N-Syn	0.9	SETD4	I396V	**T:C = 10:1**	**All_C**
19	5858876	T	C	N-Syn	1	ERAL1	M396V	**All_T**	**T:C = 1:10**
1	177772554	C	A	N-Syn	0.86	EFHA1	A220D	**C:A = 4:1**	**C:A = 1:10**
1	177772568	G	A	N-Syn	0.86	EFHA1	V225I	**All_G**	**G:A = 1:10**
13	11337736	C	G	N-Syn	0.9	GEMIN5	H283Q	**All_C**	**All_G**
5	44443744	G	G|C	N-Syn	0.75	KIAA1409	Q692H	**All_G**	**G:C = 1:10**
5	44443754	G	A	N-Syn	0.8	KIAA1409	E696K	**All_G**	**G:A = 1:4**
5	44443755	A	T	N-Syn	0.8	KIAA1409	E696V	**All_A**	**A:T = 1:4**

Ratios between reference base and SNP (Called base) in large chicken populations were bolded. FS; frameshift mutation.

### Bioinformatic analyses of genes containing amino acid change SNPs

Amino acid changes may have impacts on the functional interpretations for vitiligo induction in SL chickens. The Ingenuity Pathway Analysis (IPA) program generated bioinformatics data sets including functional groups (gene ontology; GO) and gene networks for genes containing amino acid changes in SL chicken. The 156 SNPs were found in 139 genes encompassing known- and unknown functions, chromosomal open reading frames, and hypothetical proteins (Additional file 2: Table S2).

#### Functional roles

Genes were categorized in 76 functional groups (Additional file 3: Table S3). Of these, six functional groups are of particular interest to autoimmune vitiligo development, including dermatological diseases/conditions, inflammatory response, inflammatory disease, immunological disease, immune cell trafficking, and infectious disease (Table 4). The functional group of genes for dermatological diseases/conditions contained the following genes: ADAMTS13 (ADAM metallopeptidase with thrombospondin type 1 motif 13); ASPM [asp (abnormal spindle) homolog, microcephaly associated; Drosophila)]; ATP6V0A2 (ATPase, H + transporting, lysosomal V0 subunit A2); BRCA2 (breast cancer 2, early onset); COL12A1 (collagen, type XII, alpha 1); GRM5 (glutamate receptor, metabotropic 5); LRP2 (low density lipoprotein receptor-related protein 2); MKI67 (marker of proliferation Ki-67); OBSCN (obscurin, cytoskeletal calmodulin and titin-interacting RhoGEF); PLAU (plasminogen activator, urokinase); RNF168 (ring finger protein 168, E3 ubiquitin protein ligase); STAB2 [stabilin 2 or FEEL2 (fasciclin EGF-like, laminin-type EGF-like, and link domain-containing scavenger receptor 2)]; and XIRP1 (xin actin-binding repeat containing 1). General and dermatological disease related functions for these genes are summarized in Table 5.

**Table 4:** **Vitiligo related functions of candidate genes**

Functions	# molecules	Genes involved
Dermatological diseases/conditions	14	ADAMTS13, ASPM, ATP6V0A2, BRCA2, COL12A1, GRM5, LRP2, MKI67, OBSCN, PLAU, RNF168, STAB2, XIRP1
Inflammatory response	10	ADAMTS13, CBS, OL3A1, LRP2, LRP8, NR2C2, PDE7B, PLAU, PON2, SLC6A4
Inflammatory disease	9	CBS, LRP2, MKI67, NCF2, NR2C2, PDE7B, PLAU, SLC6A4
Immunological disease	5	BRCA2, LRP2, NR2C2,PRKDC, RNF168
Immune cell trafficking	2	CBS, PLAU
Infectious disease	1	PLAU

**Table 5:** **Function of candidate genes containing SNPs in CDS region related to dermatological diseases/conditions**

Sub-groups	Molecules
Malignant cutaneous melanoma cancer	OBSCN [15]
	- Is a RhoGEF protein that interacts with cytoskeletal calmodulin and titin and is part of the giant sarcomeric signaling protein family of myosin light chain kinases
	- Mutant human OBSCN protein (E4574K) is associated with melanoma in human.
	STAB2 (or FEEL2) [16, 17]
	- is a large, transmembrane receptor protein which may function in angiogenesis, lymphocyte homing, cell adhesion, or receptor scavenging.
	- Somatic missense homozygous mutant human STAB2 gene (c.3862 T > G translating to p.S1288A) is associated with melanoma in skin from human leg (observed in 2 of 2 samples)
	LRP2 (or megalin) [17, 18]
	- Is a member of the low density lipoprotein (LDL) receptor gene family essential for brain development.
	- Somatic missense heterozygous mutant human LRP2 gene (c.6284G > A translating to p.R2095Q) is associated with melanoma in skin from human leg (observed in 2 of 2 samples).
	ASPM [17, 19]
	- This gene is the human ortholog of the Drosophila melanogaster ’abnormal spindle’ gene (asp), which is essential for normal mitotic spindle function in embryonic neuroblasts.
	- Somatic nonsense heterozygous mutant human ASPM gene (c.7174C > T translating to p.Q2392*) is associated with melanoma in skin from human leg (observed in 2 of 2 samples).
	GRM5 [20]
	- Is a member of G protein-coupled receptor that are widely expressed in the brain and modulate many diverse signaling pathways
	- In mouse melanocytes, transgenic rat GRM5 protein (S901A mutant) affects development of melanoma in mouse.
	BRCA2 [21, 22]
	- At the cellular level, loss of BRCA2 function results in sensitivity to cross-linking agents, a decrease in homology-directed repair of double-stranded DNA breaks (DSBs), and defects in replication and checkpoint control
	- Inherited mutations in BRCA1 and this gene, BRCA2, confer increased lifetime risk of developing breast or ovarian cancer.
	- Mutant human BRCA2 gene is associated with malignant melanoma in Homo sapiens.
	XIRP1 [17, 23]
	- Its function is unknown, but it is upregulated in wounded skeletal muscle cells in zebrafish
	- Somatic nonsense heterozygous mutant human XIRP1 gene (c.2838G > A translating to p.W946*) is associated with melanoma in skin from human leg (observed in 2 of 2 samples).
RIDDLE syndrome	RNF168 [24]
	- Is an E3 ubiquitin ligase
	- Mutant human RNF168 gene (deletion c.1323_1326del of ACAA and DNA duplication mutation) is associated with RIDDLE (radiosensitivity, immunodeficiency, dysmorphic features, and learning difficulties) syndrome, which a novel human immunodeficiency disorder associated with defective double strand break repair.
Schulman-Upshaw syndrome	ADAMTS13 [25]
	- Is von Willebrand Factor (VWF) cleaving metalloproteinase.
	- Is associated with the development of thrombotic thrombocytopenic purpura (TTP), known as the Schulman-Upshaw syndrome.
	- Mutant human ADAMTS13 gene (deletion c.1783_1784delTT) is associated with congenital TTP.
Autosomal recessive cutis laxa type IIA	ATP6V0A2 [26]
	- Is a subunit of the vacuolar ATPase (v-ATPase), a heteromultimeric enzyme that is essential for the acidification of diverse cellular components.
	- Mutations in human ATP6V0A2 protein (p.Q765* (rs80356758), p.R63* (rs80356750), deletion, and insertion) is associated with autosomal recessive cutis laxa type IIA.
Wrinkly skin syndrome	ATP6V0A2 [26]
	- Mutant human ATP6V0A2 gene (g.10132G > A) is associated with wrinkly skin syndrome
Hyperpigmentation	GRM5 [20]
	- In mouse melanocytes, transgenic rat mGlur5 [GRM5] protein increases hyperpigmentation of pinna in mouse ear.
Development of blister	PLAU [27, 28]
	- Is a serine protease involved in degradation of the extracellular matrix and possibly tumor cell migration and proliferation.
	- Heterozygous- and heterozygous mutant mouse Plg gene in mouse affects development of blister in mouse subepidermal skin that is altered by transgenic uPAR (PLAUR) protein and transgenic uPA (product of PLAU) protein and development of blister.

Interestingly, a recent report by Nikolaev et al. (2012) [17] indicated that amino acid changes found in ASPM, LRP2, STAB2, and XIRP1 proteins were associated with human melanoma by exome sequencing. Melanocytes in vitiligo also exhibit morphological and biological melanocyte defects/alterations compared to melanocytes from individuals with normal pigmentation [29]. While these alterations may be different from those observed in melanoma, e.g. slower growth, and higher sensitivity to oxidative stress of cultured melanocytes [30, 31], alterations in amino acid sequences found in homolog proteins but different residues may result in opposite phenotypes of dermatological diseases/conditions [32]. In addition to these molecules, BRCA2, GRM5, MKI67, and OBSCN associated with SLV are also known to be associated with human melanoma. The relationship between candidate genes and other dermatological diseases including melanoma is summarized in Table 5.

#### Gene networks

**Figure 3:**
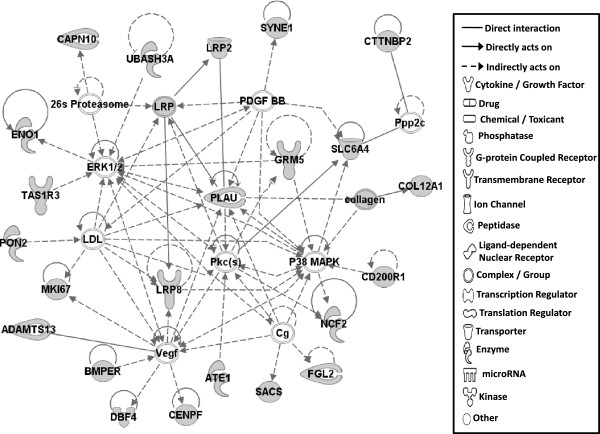
**Gene network #1.** Molecular interactions among important focus molecules are displayed. Gray symbols show the genes found in the list of SNP while white symbols indicate neighboring genes that are functionally associated, but not included, in the gene list of SNP. Symbols for each molecule are presented according to molecular functions and type of interactions.

**Figure 4:**
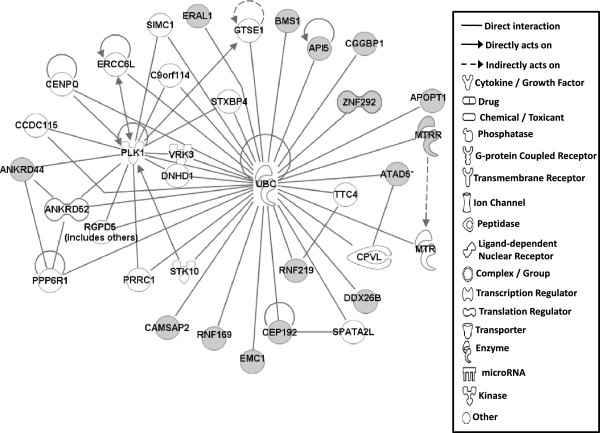
**Gene network #2.** Molecular interaction and symbols are the same as the description in Figure 3.

**Figure 5:**
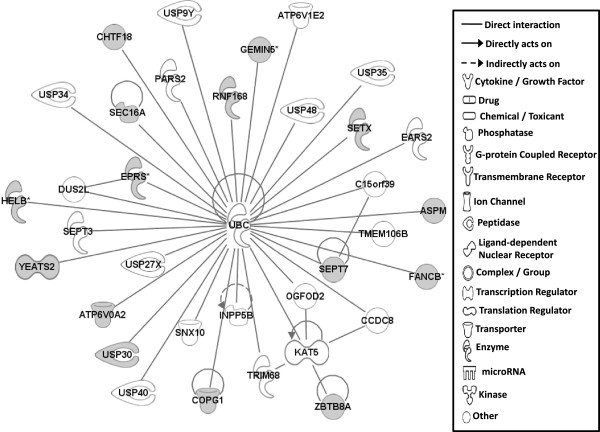
**Gene network #3.** Molecular interaction and symbols are the same as the description in Figure 3.

**Figure 6:**
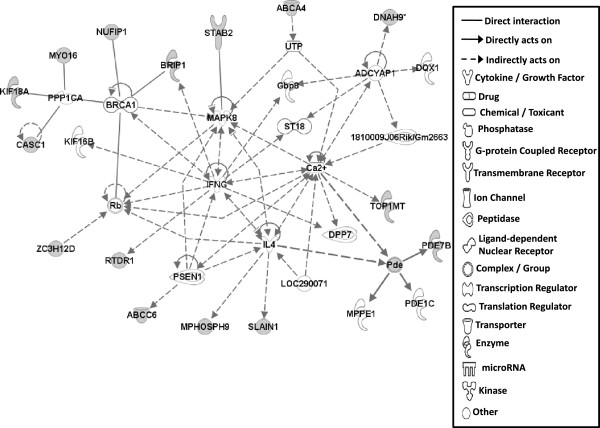
**Gene network #4.** Molecular interaction and symbols are the same as the description in Figure 3.

**Figure 7:**
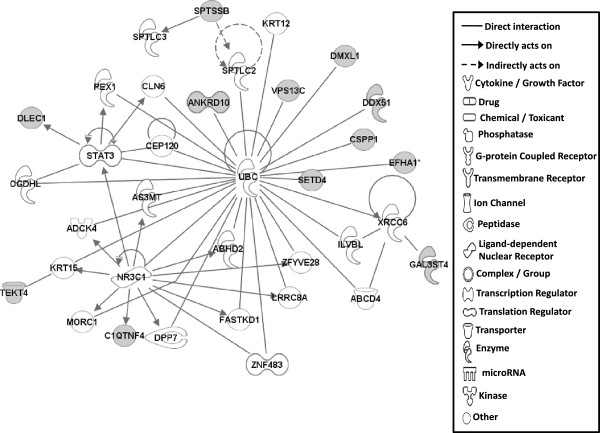
**Gene network #5.** Molecular interaction and symbols are the same as the description in Figure 3.

**Figure 8:**
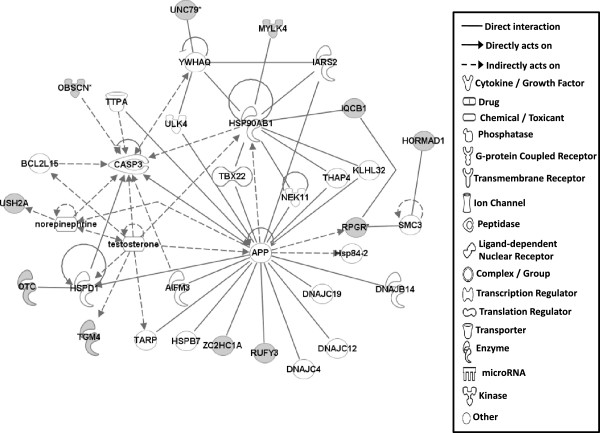
**Gene network #6.** Molecular interaction and symbols are the same as the description in Figure 3.

**Figure 9:**
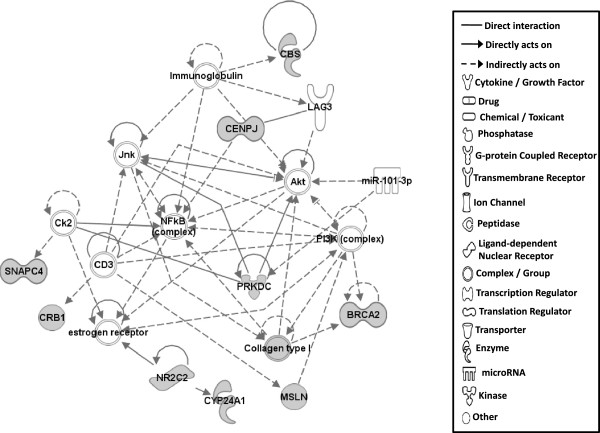
**Gene network #7.** Molecular interaction and symbols are the same as the description in Figure 3.

Gene network analysis, which represents the intermolecular connections among interacting genes based on functional knowledge inputs, was performed on genes with amino acid changes in SLV chickens using the IPA program. The gene network analysis was carried out using the simplest setting of 35 focus molecules to facilitate and summarize the intermolecular connections (Table 6 and Figures 3, 4, 5, 6, 7, 8, and 9). A discussion of the 7 gene networks is provided below and gene information for focus molecules in each network is listed in Additional file 4: Table S4.

**Table 6:** **Associated network functions of candidate genes**

ID	Molecules in network	Score	Focus molecules	Top functions
1	26 s Proteasome, ADAMTS13, ATE1, BMPER, CAPN10, CD200R1, CENPF, Cg, COL12A1, collagen, CTTNBP2, DBF4, ENO1, ERK1/2, FGL2, GRM5, LDL, LRP2, LRP8, LRP, MKI67, NCF2, P38 MAPK, PDGF BB, Pkc(s), PLAU, PON2, Ppp2c, SACS, SLC6A4, SYNE1, TAS1R3, UBASH3A, Vegf	48	24	Cardiovascular Disease, Hematological Disease, Cardiac Infarction
2	ANKRD44, ANKRD52, API5, APOPT1, ATAD5, BMS1, C9orf114, CAMSAP2, CCDC115, CENPQ, CEP192, CGGBP1, CPVL, DDX26B, DNHD1, EMC1, ERAL1, ERCC6L, GTSE1, MTR, MTRR, PLK1, PPP6R1, PRRC1, RGPD5 (includes others), RNF169, RNF219, SIMC1, SPATA2L, STK10, STXBP4, TTC4, UBC, VRK3, ZNF292	26	15	Cell Cycle, DNA Replication, Recombination, and Repair, Developmental Disorder
3	ASPM, ATP6V0A2, ATP6V1E2, C15orf39, CCDC8, CHTF18, COPG1, DUS2L, EARS2, EPRS, FANCB, GEMIN5, HELB, INPP5B, KAT5, OGFOD2, PARS2, RNF168, SEC16A, SEPT3, SEPT7, SETX, SNX10, TMEM106B, TRIM68, UBC, USP30, USP34, USP35, USP40, USP48, USP27X, USP9Y, YEATS2, ZBTB8A	26	15	Developmental Disorder, Endocrine System Disorders, Hereditary Disorder
4	1810009J06Rik/Gm2663, ABCA4, ABCC6, ADCYAP1, BRCA1, BRIP1, Ca2+, CASC1, DNAH9, DPP7, DQX1, Gbp8, IFNG, IL4, KIF16B, KIF18A, LOC290071, MAPK8, MPHOSPH9, MPPE1, MYO16, NUFIP1, Pde, PDE1C, PDE7B, PPP1CA, PSEN1, Rb, RTDR1, SLAIN1, ST18, STAB2, TOP1MT, UTP, ZC3H12D	26	15	Cell Morphology, Cellular Function and Maintenance, Hair and Skin Development and Function
5	ABCD4, ABHD2, ADCK4, ANKRD10, AS3MT, C1QTNF4, CEP120, CLN6, CSPP1, DDX51, DLEC1, DMXL1, DPP7, EFHA1, FASTKD1, GAL3ST4, ILVBL, KRT12, KRT15, LRRC8A, MORC1, NR3C1, OGDHL, PEX1, SETD4, SPTLC2, SPTLC3, SPTSSB, STAT3, TEKT4, UBC, VPS13C, XRCC6, ZFYVE28, ZNF483	20	12	Lipid Metabolism, Small Molecule Biochemistry, Digestive System Development and Function
6	AIFM3, APP, BCL2L15, CASP3, DNAJB14, DNAJC4, DNAJC12, DNAJC19, HORMAD1, Hsp84-2, HSP90AB1, HSPB7, HSPD1, IARS2, IQCB1, KLHL32, MYLK4, NEK11, norepinephrine, OBSCN, OTC, RPGR, RUFY3, SMC3, TARP, TBX22, testosterone, TGM4, THAP4, TTPA, ULK4, UNC79, USH2A, YWHAQ, ZC2HC1A	17	11	Hereditary Disorder, Ophthalmic Disease, Neurological Disease
7	Act1, Akt, BRCA2, CBS, CD3, CENPJ, Ck2, Collagen type I, CRB1, CYP24A1, estrogen receptor, Immunoglobulin, INSRR, Jnk, LAG3, miR-101-3p (and other miRNAs w/seed ACAGUAC), MSLN, MZB1, NFkB (complex), NKTR, NR2C2, PI3K (complex), PIK3IP1, PIK3R6, PRKDC, Prl4a1, Psg16, SEC14L2, SNAPC4, Taok2, Tnfrsf22/Tnfrsf23, TRAF1-TRAF2-TRAF3, VTCN1, Zfp110/Zfp369, ZNF675	14	9	Cellular Response to Therapeutics, Cellular Assembly and Organization, DNA Replication, Recombination, and Repair

Functions associated with 7 networks are listed. Score means the number of network eligible molecules out of differentially expressed genes.

Candidate genes in Network #1 are associated with signaling pathways of the mitogen activated protein kinase (MAPK; also ERK1/2) and protein kinase C (Pkc) connected to VEGF (vascular endothelial growth factor) and PDGF (platelet derived growth factor) with PLAU in the center (Figure 3). The top functions related to network #1 are cardiovascular disease, hematological disease, and cardiac infarction. Interestingly, molecules including LRP2, PLAU, ADAMTS13, and GRM5 that are part of Network #1 were also identified as functional factors for melanoma as described above [17]. Additionally, mutations in the amino acid sequence of LRP2, altered function of MAP2K1 and MAP2K2 induced by genetic mutations in melanoma patients [17], and mutations in GRM5 in mouse melanoma models [20] were also reported. The connections in Network #1 therefore suggest genetic mutations that generated amino acid changes in LRP2, PLAU, ADAMTS13, and GRM5 may influence dermatological diseases, including vitiligo, through MAPK and ERK1/2 signaling pathways.

The top functions of Network #2 include Cell Cycle, DNA Replication, Recombination and Repair, and Developmental Disorder (Figure 4) and Network #3 is involved in Developmental Disorder, Endocrine System Disorders, Hereditary Disorder (Figure 5). Most molecules in Networks #2 and #3 directly bind to UBC (ubiquitin C). Ubiquitinylation, the covalent attachment of ubiquitin to proteins, regulates numerous cellular processes such as protein degradation and signal transduction. Recently, many ubiquitinylated proteins and their lysine ubiquitinylation site were identified using proteomic technologies in mammalian species [33–37]. Indeed, in SLV, the amino acid changes in lysine residues for CEP192 (centrosomal protein 192 kDa; pK169R) and API5 (apoptosis inhibitor 5; pK1219Q) were identified (Table 2), suggesting that the various cellular functions including protein degradation by altered ubiquitinylation properties of proteins may play a significant role in the induction of vitiligo.

Molecules in Network #4 are involved in Cell Morphology, Cellular Function and Maintenance, Hair and Skin Development and Function (Figure 6). Molecules in network #4 mainly interact with IFNG (interferon gamma), IL4 (interleukin 4), MAPK8, and calcium signaling pathways in addition to protein phosphatase (PPP1CA; protein phosphatase 1 catalytic subunit alpha isozyme) functions. MYO16 (myocin 16), KIF18A (kinesin family member 18A), and CASC1 (cancer susceptibility candidate 1) are known to directly bind to PPP1CA [38–40], suggesting that amino acid changes in those proteins may induce alterations (hyper vs hypo) in protein phophorylation states in SL chickens, resulting in vitiligo development. Molecules interacting with IFNG, IL4, MAPK8 and calcium signaling pathways showed an indirect relationship, not a direct relationship with each other making it difficult to explain how amino acid changes in these molecules affect vitiligo induction in SL chicken.

Molecules in Network #5 also mainly bind to UBC as discussed in Networks #2 and #3 and the functions include Lipid Metabolism, Small Molecule Biochemistry, Digestive System Development and Function (Figure 7).

Network #6 contains molecules involved in Hereditary Disorder, Ophthalmic Disease, Neurological Disease (Figure 8). In this network, ZC2HC1A (zinc finger, C2HC-type containing 1A) and RUFY3 (RUN and FYVE domain containing 3) directly bind to APP [amyloide beta (A4) precursor protein]. APP is a precursor protein for beta-amyloide, which is the main constituent of amyloid plaques in the brains of Alzheimer disease patients [41]. RUFY3 (also known as single axon-related; singar1), which is a brain specific protein, regulates neuronal polarity by suppressing formation of surplus axons [42]. Though the binding of ZC2HC1A and RUFY3 to APP was found during the progression of Alzheimer disease [41], the functional roles for this binding in the progression of this disease has not been characterized. Similarly, amino acid changes found in ZC2HC1A and RUFY3 are implicated in SLV development possibly as a result of altered APP binding properties. USH2A [Usher syndrome 2A (autosomal recessive, mild)] was included in network #6. Various mutations in USH2A have been identified in patients of Usher syndrome type II, which is characterized by moderate to severe sensorineural hearing loss and progressive retinitis pigmentosa [43]. Vitiliginous SL chickens may also develop severe visual impairment and blindness due to autoimmune activity directed against choroidal melanocytes and subsequent damage to the retinal pigment epithelium. [44, 45]. Taken together, the amino acid change in USH2A also may affect vitiligo progression and retinal depigmentation.

Molecules in Network #7 mainly function in Cellular Response to Therapeutics, Cellular Assembly and Organization, DNA Replication, Recombination, and Repair. PRKDC (protein kinase, DNA-activated, catalytic polypeptide) and its interacting protein kinases are mainly involved in this network (Figure 9). In addition to knock-out and inactive mutations, alteration of autophosphorylation capability by single amino acid change of PRKDC has been known to influence rejoining of DNA double stranded breaks in mammalian cells [46], suggesting that vitiligo development may be affected by aberrant PRKDC kinase activity due to an observed amino acid change.

Vitiligo susceptible loci in human populations identified by several GWAS [5–10] also showed several loci that induced amino acid changes in various proteins such as STRN3 (Striatin, calmodulin binding protein 3), DNAH5 (dynein, axonemal, heavy chain 5), KIAA1005 (immunoglobulin heavy variable 3), TYR (tyrosinase), OCA2 (oculocutaneous albinism II), PTPN22 [protein tyrosine phosphatase, non-receptor type 22 (lymphoid)], IFIH1 (interferon induced with helicase C domain 1), SLA (SRC-like-adaptor), CD44, MC1R [melanocortin 1 receptor (alpha melanocyte stimulating hormone receptor)], UBASH3A (ubiquitin associated and SH3 domain containing A), C1QTNF6 (C1Q and tumor necrosis factor related protein 6), CASP7 (caspase 7), and GZMB (granzyme B). One similar mutation in UBASH3A protein coding region from human [6] was also found in the SLV chicken model (Table 2). Additionally, when the long list of 3518 candidate amino acid altering SNPs (read depth <10) was considered, several genes, including IFIH1 (interferon-induced helicase C domain-containing protein 1), CD44 antigen and DNAH5 (dynein, axonemal, heavy chain 5), matched those identified by human GWAS studies [Additional file 1: Table S1 and [5, 6]], although the amino acid position and alterations did not match. UBASH3A is one of the two family members belonging to the T cell ubiquitin ligand (TULA) family and can negatively regulate T cell signaling [47]. Together with the UBC molecule discussed elsewhere in this paper, functions for UBASH3A related to ubiquitinylation and T cell signaling pathway may be important for SLV development. IFIH1 encodes an interferon-induced RNA helicase involved in antiviral innate immune responses, associated with type 1 diabetes, Graves’ disease, multiple sclerosis, psoriasis, and perhaps lupus [48–53]. CD44 encodes a cell surface glycoprotein with various functions, including a role in T cell development [54], and is associated with lupus [55]. DNAH5 gene mutation is found in patients with primary ciliary dyskinesia (PCD) [56], a rare disease transmitted as an autosomal recessive trait and characterized by recurrent airway infections due to abnormal ciliary structure and function. Primary defects in the structure and function of sensory and motile cilia result in multiple ciliopathies [57].

## Conclusions

In this study, various potential genetic markers showing amino acid changes were identified in the SLV model through genome re-sequencing. When considering functionality based on the interpretation of factors involved, development of vitiligo appeared to be associated with the interactions among cytoskeletal factors (OBSCN, ASPM, XIRP1, ADAMTS13), protein kinases (MAPK, ERK1/2, PKC, PRKDC), phosphatase (PPP1CA), ubiquitinylation (UBC) and amyloid (APP) production. Further functional validation study, such as allele specific expression of the candidate genes with candidate SNPs at the target tissues involved in SLV development will be carried out using the SL chicken model for spontaneous autoimmune vitiligo.

## Methods

### Animals and Illumina sequencing

Adult SL chickens with vitiligo and parental non-vitiliginous BL chickens, maintained by G. Erf at the University of Arkansas (Fayetteville, AR), were selected from the breeder populations. Blood (3 ml) was collected from 12 birds each following an animal use protocol approved by the University of Arkansas Institutional Animal Care and Use Committee (IACUC; approval number: 11019). Genomic DNA was isolated from each whole blood sample using QiaAmp DNA mini kit (Qiagen, Hilden, Germany) following manufacturer’s instructions. DNA quality was determined by agarose gel electrophoresis and 10 samples having the highest quality in each line were pooled to represent each line. Library preparation and Illumina genome sequencing for the pooled DNA samples were performed by the National Center for Genome Resources (NCGR; Santa Fe, NM). Illumina HiSeq system 2x100 bp paired end read technology was used for genome sequencing.

### Genome sequence assembly and data analysis

Illumina sequencing data received from NCGR was aligned to the chicken reference genome sequence for Red Jungle Fowl (GBK 4.0) that was retrieved from NCBI. For the reference based genome alignment, the NGen genome sequence assembly program of the Lasergene software package (DNAStar, Madison, WI) was used. Assembly parameters were as follows: file format, BAM; mer Size, 21; mer skip query, 2; minimum match percentage, 93; maximum gap size, 6; minimum aligned length, 35; match score, 10; mismatch penalty, 20; gap penalty, 30; SNP calculation method, diploid bayesian; minimum SNP percentage, 5; SNP confidence threshold, 10; minimum SNP count, 2; minimum base quality score, 5. After assembly, the SeqMan Pro program of the Lasergene package was used for further analyses including SNP data.

### SNP detection and analysis

JMP genomics (SAS Institute, Inc., Cary, NC) program was used for filtering unique SNPs for vitiligo SL chickens. SNPs occurring in both SL and BL lines were filtered out, leaving behind unique SNPs for each line. To identify highly fixed and homozygous SNPs, the SNPs were filtered based on SNP percentages (SNP%). SNPs with a SNP% of ≥75 (%) (for example, number of SNP = 3 of read depth = 4) were chosen. The 75% cutoff for SNP selection was set by considering potential sequencing errors that can be generated by the massively parallel sequencing method. Potential causal SL SNPs that induce non-synonymous changes in CDS regions were chosen for further analysis. Since the read depth of many SL SNPs was low, unique SNPs showing ≥10 read depths were considered as reliable SNPs. Reliable and causal SNPs, which were chosen by criteria described above were confirmed by double-checking the raw assembly data with alignment view to reduce false positives.

### SNP validation using PCR and Sanger sequencing

Fourteen randomly chosen SNPs, which induce amino acid changes in the CDS region, were subjected to validation using PCR and Sanger sequencing with larger numbers of SL and BL chickens. Twenty BL and 70 SL chickens that were verified phenotypically to be non-vitiliginous and vitiliginous, respectively, were used for blood sampling. Approximately 100 μL of blood was collected from each bird by wing vein puncture into tubes containing citrate (anticoagulant). Genomic DNA was isolated from whole blood using the Wizard SV 96 Genomic DNA Purification System (Promega; Madison, WI) following manufacturer’s instructions with modifications. Isolated DNA was quantified using a Nanodrop 1000 spectrophotometer (Thermo Fisher Scientific Inc., Waltham, MA) and a dilution of 1 ng/μL was prepared in 96 well PCR format for all samples. For PCR reaction, forward and reverse primers were designed based on the RJF genome sequence (GenBank assembly ID: GCA_000002315.2) using Primer 3 online software (Table 7). The sequencing primers were designed to anneal at least 50 bp upstream of the SNP position and forward/reverse primers were chosen at the flanking regions of the seq primer and the SNP position. All primers were commercially synthesized by Integrated DNA Technology (Ames, IA). PCR was carried out as 25 μL reaction volumes in 96 well plates with cyle conditions as follows: denaturation 95°C for 1 min, 40 cycles of amplification (95°C for 30 s, 55-63°C for 1 min, 72°C for 1 min), final extension 72°C for 10 min. Verification of PCR was performed by 1% agarose gel electrophoresis. PCR products were purified using the Wizard SV 96 PCR Clean-Up System (Promega; Madison, WI) following manufacturer’s instructions. Briefly, four plates (four different PCR products) were pooled into one plate and were subjected to PCR clean-up. Cross-specificity of seq-primers to the four pooled PCR products was examined by BLAST function (NCBI) and only products that were not cross-specific with other seq primers were pooled. Purified PCR products were subjected to Sanger sequencing performed by the University of Arkansas DNA Resources Center (Fayetteville, AR). Results were analyzed using ABI Sequence scanner software (Life Technologies, Carlsbad, CA). Ratios of bases occurring at SNP locations were recorded.

**Table 7:** **Primers used for PCR and Sanger sequencing**

Gene	Primer name	Primer sequence (5′→3′)
EFHA1	Forward	CAAAAACCTAAATGGGTTTCCA
	Reverse	AAACTTCATCAGGACATGCAGA
	Sequencing	GTGCAAGTTTCTGAAAGACT
EPRS	Forward	CAATTCCACACTTTGCAGGTTA
	Reverse	GCTGTGATGCCAAATTTAAACA
	Sequencing	GAAGGGAAGGCATATGTGGA
ERAL1	Forward	AGGACACACACATGCTGGATAC
	Reverse	GCCCTTTTTGTGTTTTAAGTGG
	Sequencing	GAGACTTCCTTGGGACCACA
LOC100859401	Forward	GAATTTACCAGTCCAGGCACTC
	Reverse	ACTACCTTGGGCCTTGTCAGTA
	Sequencing	ATGCTCCTTTTTTCCAGACC
LOC770458	Forward	TGCAGAGAATACAGCACGATCT
	Reverse	ACTCACTCCATAAGGGGAGACA
	Sequencing	GCCTTTACCAGACAAA
SPTSSB	Forward	CTTGTTGGGAATCAGCTCTCTT
	Reverse	TGCCTTTGTCAATACTGTGACC
	Sequencing	AACGCAGAGTCCTAACGTGG
STAB2	Forward	CACTGTTACTGCAGTGATGCAA
	Reverse	GATAGGAACAGCATCCCTAACG
	Sequencing	GCACTGGCACGTGTTGTTCT
SYNE1	Forward	ACTCACCTTGTGGTTGGCTAGT
	Reverse	CCTCACTGTCTTCCTCTGCT
	Sequencing	CCAGCCTGCTAGACATATGT
SETD4	Forward	CAAATCGTTGTCACGTTCAAGT
	Reverse	TTCCTTTTGGTGTGCTTCCTAT
	Sequencing	AGATCTTCCA AGCGTAGTGC
GEMIN5-1	Forward	TAGGCTTCATTTGCTGACTCAA
	Reverse	TACAGCAGGAAGGAAGGATGAT
	Sequencing	CCCTTTCTTTTCCAAAGGTG
GEMIN5-2	Forward	CCTGGGATGAGGTAGTGAAAAG
	Reverse	AAGCAGAAAAGCAAAAGCAGAC
	Sequencing	ATTCTTGGTGCTGTGGCCCG
KIAA1409_1	Forward	CTTTGGGCTTAGAGAACAGCAT
	Reverse	AAATTCAGTGGCATTTTTGCTT
	Sequencing	GGGGTGGTTCTCACACATGTCA

### Bioinformatics

Functional interpretation of 139 genes showing ≥75 SNP%, ≥10 read depths and non-synonymous changes was analyzed in the context of gene ontology and molecular networks using Ingenuity Pathways Analysis (IPA; Ingenuity Systems®; http://www.ingenuity.com). Since IPA is based on human and mouse bioinformatics, functionalities for differentially expressed genes in the chicken were interpreted based primarily on mammalian biological mechanisms. The limit of number of molecules in the network was set to 35, leaving only the most important molecules based on the number of connections for each focus gene (a subset of uploaded significant genes having direct interactions with other genes in the database) to other significant genes [58].

### Availability of supporting data

All sequence reads described in the manuscript are available under BioProject accession PRJNA256208. Illumina sequence reads have been deposited at NCBI’s SRA archive under following numbers (SL: Sample: SRS670088, Experiment: SRX665272, Read: SRR1531502; BL: Sample: SRS670098, Experiment: SRX665286, Reads: SRR1531503).

## Electronic supplementary material

Additional file 1: **List of 3518 SNPs to induce amino acid changes in SL chicken.** (XLSX 432 KB)

Additional file 2: **Gene name and functions of 139 gene containing amino acid changes showing** ≥**10 depth counts in SL chicken.** Gene symbol, Entrez gene name, cellular location and type were provided. (XLSX 18 KB)

Additional file 3: **Functional groups of genes containing amino acid changes.** (XLSX 12 KB)

Additional file 4: **List of focus molecules in gene networks.** Gene symbols and Entrez gene names were displayed for the illustrations of network analysis. (XLSX 17 KB)
